# T-Cell/Histiocyte-Rich Large B-Cell Lymphoma Presented as T-Lymphoid Hyperplasia Involving the Central Nervous System

**DOI:** 10.7759/cureus.1119

**Published:** 2017-03-26

**Authors:** Mayumi Kubota, Makoto Taniguchi, Shinsuke Tobisawa, Yasuhiro Nakata, Muneo Nakaya, Hiroyuki Tamogami, Manabu Matsunawa, Takashi Komori

**Affiliations:** 1 Department of Neurosurgery, Tokyo Metropolitan Tama Medical Center; 2 Department of Neurosurgery, Tokyo Metropolitan Neurological Hospital; 3 Department of Neurology, Tokyo Metropolitan Neurological Hospital; 4 Department of Neuroradiology, Tokyo Metropolitan Neurological Hospital; 5 Department of Otolaryngology-Head and Neck Surgery, Tokyo Metropolitan Tama Medical Center; 6 Department of Hematology, Tokyo Metropolitan Tama Medical Center; 7 Division of Hematology and Oncology, Department of Internal Medicine, St. Marianna University School of Medicine; 8 Department of Neuropathology, Tokyo Metropolitan Neurological Hospital

**Keywords:** secondary cns lymphoma, lymphomatoid granulomatosis, lymphoproliferative disorders, t-cell hyperplasia

## Abstract

We herein report a case of T-cell/histiocyte-rich large B-cell lymphoma which initially presented as a self-limiting T-lymphoproliferative disorder involving multiple extranodal and extrapulmonary organs, such as the salivary gland, the liver, and the central nervous system. Repeated biopsies only revealed polyclonal T-lymphocytosis without the presence of atypical B-cells. Angiocentric cellular infiltration was absent, thus ruling out lymphomatoid granulomatosis. A recurrence in the lymphatic system finally revealed a small population of pathognomonic atypical B-cells, which led to the diagnosis. The clinical dilemma in the diagnosis and management of this indeterminate condition points to limitations in the current nosology.

## Introduction

Lymphocytic proliferation results from either monoclonal proliferation of neoplastic lymphoma cells or polyclonal proliferation as a normal immune response [1]. A dysregulated or exaggerated response can destroy tissue and impair organ function. Triggers for the activation of cell-mediated immunity are usually microorganisms, such as Epstein-Barr virus (EBV), or possibly neoplastic cells, as seen in paraneoplastic syndromes [2-3]. With the advancement of immunosuppressive therapy and molecular diagnostic techniques, iatrogenic lymphoproliferative disorder (LPD) in immunodysregulated patients are beginning to be recognized as an emerging medical problem today. We herein report a case of possibly iatrogenic LPD, which required more than two years from the initial presentation until confirmation of malignancy.

## Case presentation

A 66-year-old woman with a history of rheumatoid arthritis was referred to our hospital after hospitalization at a nearby general care facility for altered mental status and weakness. She had developed a parotid tumor 18 months before while undergoing disease modifying anti-rheumatoid therapy with methotrexate. The tumor was surgically removed and diagnosed as benign T-cell proliferation. The methotrexate was replaced with a monoclonal antibody at that time, and the patient developed fever, fatigue, and hepatosplenomegaly shortly thereafter. A liver biopsy again demonstrated benign T-cell proliferation despite clinical suspicion of infectious mononucleosis. The monoclonal antibody (Tocilizumab) was discontinued, and dexamethasone was administered until clinical remission. Her physician had conducted tests for malignant lymphoma and infectious mononucleosis, all of which were negative. Systemic manifestations were not detected in the radiological findings or by repeated bone marrow analyses. After a disease-free period of eight months, she started to complain of fatigue and weakness and rapidly became bedridden.

When the patient was transferred to our hospital, she was weak and drowsy, and the neurological assessment was remarkable for spontaneous nystagmus and severe quadriparesis with positive pyramidal signs. She was normothermic without signs of meningeal irritation. Brain magnetic resonance imaging (MRI) demonstrated multiple lesions in the central nervous system (CNS). She was admitted for further evaluations and treatment as described below.

Hematological results were normal without any blast cells. A soluble IL-2 receptor was 473 U/mL in the serum (reference range: 122-496 U/mL) and 103 U/mL in the cerebrospinal fluid (CSF). Her CSF showed pleocytosis of the lymphocytes (18 × 10^6^/L), elevated concentration of proteins (0.10 g/dL), immunoglobulins (IgG 21.8 mg/dL, IgM 1.4 mg/dL), and normal glucose concentration (64 mg/dL). Her serum was positive for rheumatoid factor (18 U/mL), anti-thyroperoxidase antibody (404 IU/mL), and anti-cardiolipin beta-2 glycoprotein I antibody (5.4 U/mL). Infectious workups were nonspecific for serum or CSF. Cytological analysis of the CSF revealed no atypical cells.

**Figure 1:**
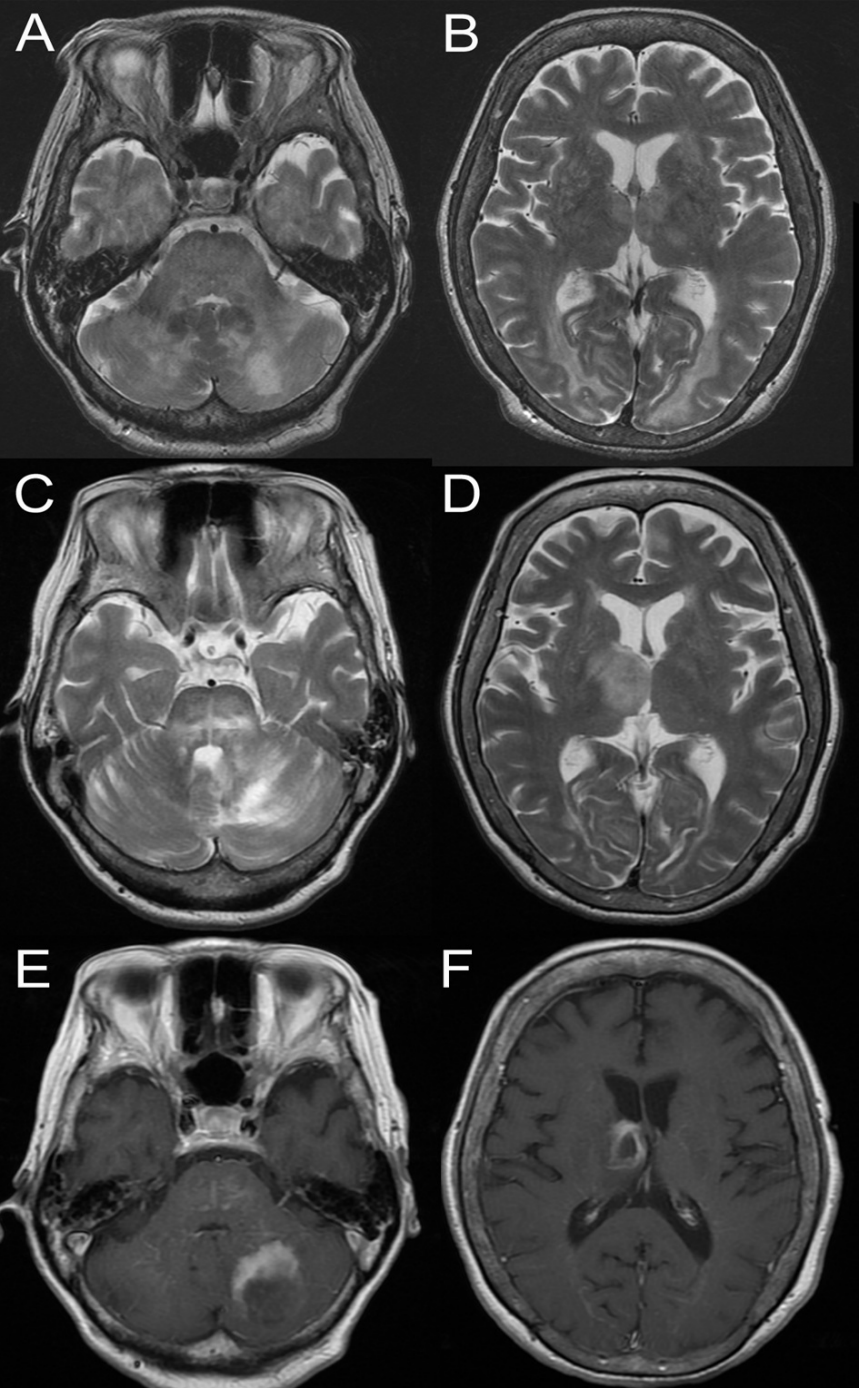
Brain magnetic resonance imaging of the patient. A-B, T2-weighed imaging at initial presentation: (A) Hyperintense signal in the bilateral cerebellar hemispheres; (B) Hyperintensity noted in the bilateral thalamus and bioccipital subcortex; C-F, T2-weighted imaging and T1-weighted imaging with gadolinium enhancement immediately before brain biopsy; (C) Hyperintense lesions noted in the pons and the left side of the cerebellum; (D) Progression of the lesion of the thalamus on the right; (E) Ring-enhancing lesion of the cerebellum; (F) Ring-enhancing lesion of the thalamus.

Brain MRI demonstrated an increased T2 signal at multiple sites in the brain parenchyma, such as the left cerebellar hemisphere, the brainstem, and the right thalamus, some of which were swollen and enhanced with gadolinium (Figures 1A-1F). No lesion was demonstrated in the cervical spinal cord MRI. Computed tomography (CT) scanning of the body did not reveal any massive lesions in the lung, the solid abdominal organs, or the lymph nodes. Malignant lymphoma, lymphomatoid granulomatosis (LYG), neurosarcoidosis, and neuroinflammatory response caused by an unidentified opportunistic infection were suspected.

Empirical treatment with methylprednisolone had only suboptimal effects. Repeated brain MRI revealed growth of the lesion in the right thalamus while the lesions in the limbic lobes subsided. Another course of methylprednisolone failed to improve her neurological deficit or radiological abnormality. Because the multiple lesions in the brain parenchyma were considered to be progressing as a whole, a brain biopsy was planned.

A stereotactic biopsy was performed under general anesthesia on the 98th day after admission. The tissue sections of the biopsy specimens were prepared for hematoxylin-eosin (HE) staining, periodic acid-Schiff staining, immunohistochemistry for antibodies (glial fibrillary acidic protein, cluster of differentiation 3 (CD3), CD4, CD8, CD10, CD20, CD68, CD79a, T-cell intracytoplasmic antigen-1 (TIA-1), granzyme B, latent membrane protein (LMP), Epstein-Barr virus nuclear antigen 2 (EBNA2), and Ki-67), and in situ hybridization for Epstein-Barr virus expressing mRNA (EBER). Cells in the tissue were extracted, and surface markers were analyzed by flow cytometry. The karyotype was assessed by G-banding.

**Figure 2:**
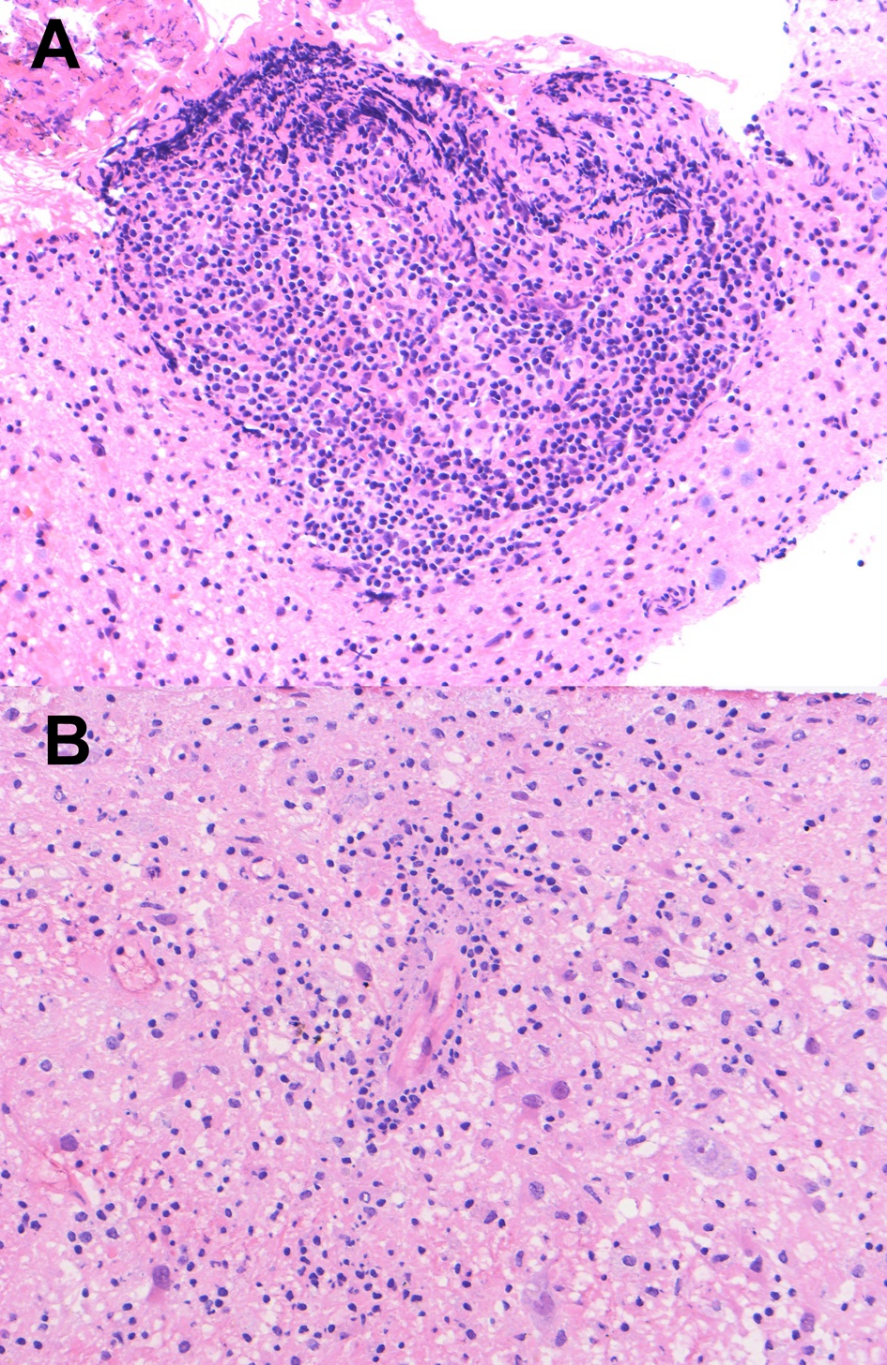
Pathological features of the lesion. (A) Hematoxylin-eosin staining of the brain biopsy specimens revealed proliferation and infiltration of small lymphocytes. (B) The blood vessel shown here is surrounded by lymphocytes. Note that the vessel walls are intact.

Pathological examination revealed aggressive infiltration of lymphocytes destroying the parenchyma, which was surrounded by a sparsely infiltrated area (Figure 2A). Infiltrating small lymphocytes were positive for CD3. The pattern of infiltration was only partially perivascular, and vessel walls were basically intact (Figure 2B). Well-formed granulomas were not observed. There was a small population of B-cells positive for CD20 or CD79a. EBER and EBNA were both negative in all of the cellular components. Neutrophils and acidophils were rarely observed. Damaged tissue contained an abundance of histiocytes, along with reactive astrocytes. MIB-1 labeling index was less than one percent.

Based on these results and the radiological findings, the authors considered the disease to be a form of T-cell lymphoproliferative disorder, in spite of some clinical manifestations resembling lymphomatoid granulomatosis. Because the natural history of this disease condition was not obvious, and the general status of the patient was not deteriorating, we refrained from initiating immediate treatment. Meanwhile, another CT scan of the extracranial regions revealed cervical lymph node swelling.

**Figure 3:**
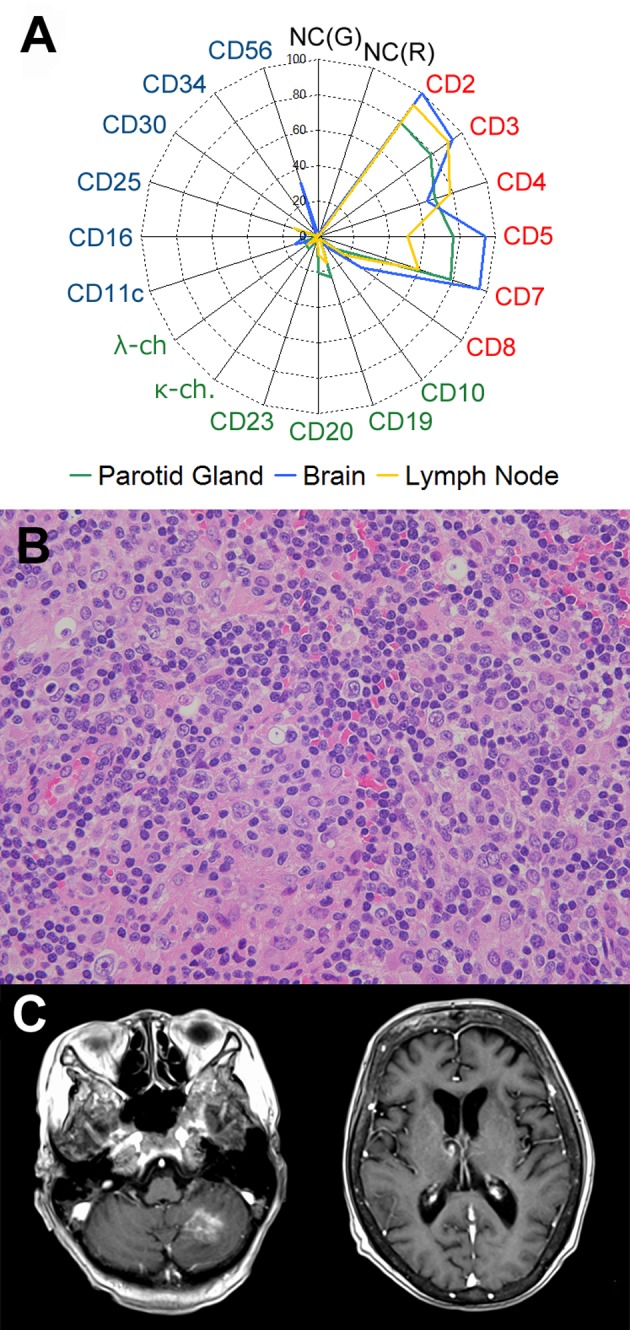
Findings from the lymph node specimen, and the follow-up CNS imaging. (A) Flow-cytometry data summarized on a radar chart. The specimens from the parotid gland, the brain, and the cervical lymph node each demonstrated proliferation of the T-cell lineage. (B) Large atypical cells are distributed sparsely against a background of lymph proliferation. (Cervical lymph node, hematoxylin-eosin stain, x 40). (C) magnetic resonance imaging after chemotherapy showed remarkable regression of the lesions.

Further, a needle biopsy of the cervical lymph node again revealed T-cell-dominant lymphatic pleocytosis. Flow cytometry demonstrated cellular components similar to those found in the other sites (Figure 3A). However, atypical large cells, which were positive for CD20, weakly positive for paired box 5 (PAX5), CD5, CD30, CD3, CD4, CD8, and TIA-1 and negative for granzyme B, were remarkably commingled with morphologically normal immune cells (Figure 3B). LMP and EBNA2 were negative, but some of the large cells were positive for EBER. The diagnosis of T-cell/histiocyte-rich large B-cell lymphoma (THRLBCL) was made based on the cellular composition and the molecular profile.

Systemic chemotherapy with rituximab, high-dose methotrexate, and cytarabine was initiated immediately after the diagnosis. Although the imaging studies demonstrated remission of the CNS lesions after two sessions of chemotherapy, the patient remained bedridden (Figure 3C).

## Discussion

We described the first case on record of THRLBCL with phasic presentations of extranodal T-lymphoid hyperplasia. Although lymphoproliferative disorders and malignant lymphomas are well-recognized comorbidities in patients under immunosuppressive treatment [4], this case is remarkable for its presentation of contradictory benign and malignant characteristics. Whereas the metastatic features of the disease implied a malignancy, several facts such as the absence of neoplastic cells in the lesions, the blood, and the CSF, and the self-limiting invasiveness seemed to favor a benign interpretation, raising the issue of whether this disorder could correctly be classified as a single nosological entity.

It has been recognized recently that several B-cell neoplasms, such as diffuse large B-cell lymphoma (DLBCL), LYG, and some forms of Hodgkin lymphomas are EBV-related [5-7]. According to Castillo, et al., DLBCL in the elderly has 100% EBER expression, and DLBCL associated with chronic inflammation, as well as 3-10% of DLBCL cases not otherwise specified, is related to EBV infection [8]. LYG lesions at Stage II and III have 100% EBER expression by definition [5]. Endemic type Burkitt’s lymphoma is also positive for EBV infection [9]. Most of these disorders result from the relapse of dormant EBV secondary to acquired or congenital immunodeficiency status.

Although it is difficult to determine how the disease progressed in this patient, two interpretations are possible. Firstly, the patient could have developed lymphoproliferative lesions like LYG, which were progressive. Dormant EBV in B-cells could have become active due to immunosuppression by low-dose methotrexate and/or the immunosenescence of the host, and the cell-mediated immune response could have been provoked by humoral factors from the EBV-positive-B-cells and/or products of EBV. The exaggerated neuroinflammatory response, not the clonal expansion of the neoplastic cells, was the primary pathology of the disease. However, pathological evidence of EBV infection in this patient was weak; EBER expression was absent in the parotid gland, liver, and brain specimens. Moreover, expression of EBER was not ubiquitous in the atypical B-cells observed in the cervical lymph node.

Secondly, the patient had developed THRLBCL with or without the influence of methotrexate, which caused multiple extranodal and nodal lesions. The location and the timing of the biopsy could have complicated the results of the CNS pathology. We selected a lesion in the right thalamus for biopsy, while the largest mass was located in the cerebellum. Furthermore, the brain biopsy was performed after two courses of high-dose corticosteroid administration because we expected that steroid therapy would prevent disease progression. Considering the fact that corticosteroids were not administered prior to the biopsies of the non-CNS lesion and that atypical B-cells were not detected in these specimens, this interpretation is less likely.

A case of non-CNS THRLBCL mimicking LYG was reported by Olivieri, et al. [10]. The previously healthy patient presented with a large abdominal mass, left-sided inguinal adenopathy, and pulmonary nodular lesions. Pathological findings were remarkable due to the proliferation of EBV-negative large B-cells intermingled with non-neoplastic T-cells and perivascular hypercellularity. The lesion had a stronger malignant profile compared to our case, probably reflecting a different background in immunocompetency. The difficulty of managing these cases points to the need to understand better which signals provoke the infiltration of T-cells.

## Conclusions

We experienced a case of secondary CNS THRLBCL whose clinical, radiological, and pathological interpretation was inconclusive until the final diagnosis. T-lymphocytic hyperplasia in the context of immunodysregulation may be responsible for occult neoplastic lymphocytes. Further understanding of the molecular pathomechanisms of these diseases will allow us to give the condition a better clinical entity, which would be the first step in order to manage them more effectively.

## References

[1] Kumar V, Abbas AK, Fausto N (2005). Robbins and Cotran Pathological Basis of Disease, 7th ed.

[2] de Beukelaar JW, van Arkel C, van den Bent MJ (2005). Resolution of EBV(+) CNS lymphoma with appearance of CSF EBV-specific T cells. Ann Neurol.

[3] Savage PA, Vosseller K, Kang C (2008). Recognition of a ubiquitous self antigen by prostate cancer-infiltrating CD8+ T lymphocytes. Science.

[4] Lake W, Chang JE, Kennedy T (2013). A case series of primary central nervous system posttransplantation lymphoproliferative disorder: imaging and clinical characteristics. Neurosurgery.

[5] Campo E, Swerdlow SH, Harris NL (2011). The 2008 WHO classification of lymphoid neoplasms and beyond: evolving concepts and practical applications. Blood.

[6] Song JY, Pittaluga S, Dunleavy K (2015). Lymphomatoid granulomatosis--a single institute experience: pathologic findings and clinical correlations. Am J Surg Pathol.

[7] DeGraaff HJ, Wattjes MP, Rozemuller-Kwakkel AJ (2013). Fatal B-cell lymphoma following chronic lymphocytic inflammation with pontine perivasucular enhancement responsive to steroids. JAMA Neurol.

[8] Castillo JJ, Beltran BE, Miranda RN (2016). EBV-positive diffuse large B-cell lymphoma of the elderly: 2016 update on diagnosis, risk-stratification, and management. Am J Hematol.

[9] Longo DL, Fauci A, Kasper D, Hauser SL, Jameson JL, Loscalzo J (2012). Harrison’s Principles of Internal Medicine, 18th ed. http://www.amazon.com/Harrisons-Manual-Medicine-18th-Longo/dp/007174519X/ref=sr_1_6?s=books&ie=UTF8&qid=1490254476&sr=1-6&keywords=Harrison%E2%80%99s+Principles+of+Internal+Medicine.

[10] Olivieri A, Sabattini E, Goteri G (2014). Fatal necrotizing angiotropic Epstein-Barr virus-negative large B-cell lymphoma: a case report with unusual clinicopathological features in-between lymphomatoid granulomatosis and T-cell/histiocyte-rich large B-cell lymphoma. Medicine (Baltimore).

